# Comparative analysis of novel and common reference genes in adult tissues of the mussel *Mytilus galloprovincialis*

**DOI:** 10.1186/s12864-022-08553-1

**Published:** 2022-05-06

**Authors:** Federica Salatiello, Marco Gerdol, Alberto Pallavicini, Annamaria Locascio, Maria Sirakov

**Affiliations:** 1 Stazione Zoologica Anton Dohrn, Department of Biology and Evolution of Marine Organisms, Villa Comunale 1, 80121 Naples, Italy; 2 Department of Life Sciences, Università degli Studi di Trieste, Via Licio Giorgieri 5, 34127 Trieste, Italy

**Keywords:** *Mytilus galloprovincialis*, Reference genes, Real time RT-PCR, RNAseq

## Abstract

**Background:**

Real-time quantitative PCR is a widely used method for gene expression analyses in various organisms. Its accuracy mainly relies on the correct selection of reference genes. Any experimental plan involving real-time PCR needs to evaluate the characteristics of the samples to be examined and the relative stability of reference genes. Most studies in mollusks rely on reference genes commonly used in vertebrates.

**Results:**

In this study, we focused on the transcriptome of the bivalve mollusk *Mytilus galloprovincialis* in physiological state to identify suitable reference genes in several adult tissues. Candidate genes with highly stable expression across 51 RNA-seq datasets from multiple tissues were selected through genome-wide bioinformatics analysis. This approach led to the identification of three genes (*Rpl14*, *Rpl32* and *Rpl34*), whose suitability was evaluated together with 7 other reference genes commonly reported in literature (*Act*, *Cyp-A*, *Ef1α*, *Gapdh*, *18S*, *28S* and *Rps4*). The stability analyses performed with geNorm, NormFinder and Bestkeeper identified specific either single or pairs of genes suitable as references for gene expression analyses in specific tissues and revealed the *Act*/*Cyp-A* pair as the most appropriate to analyze gene expression across different tissues.

**Conclusion:**

*Mytilus galloprovincialis* is a model system increasingly used in ecotoxicology and molecular studies. Our transcriptome-wide approach represents the first comprehensive investigation aimed at the identification of suitable reference genes for expression studies in this species.

**Supplementary Information:**

The online version contains supplementary material available at 10.1186/s12864-022-08553-1.

## Background

Mussels are organisms important both from a commercial and from a scientific standpoint. With a global production of several hundred thousand tons per year, *M. galloprovincialis* is one of the most relevant farmed edible bivalve in the Mediterranean area [1]. The use of mussels as model organism covers a broad range of research fields: from biomonitoring and ecotoxicology [2], to translational medicine [3], to invertebrate immunology [4]. The growing scientific interest in these organisms is progressively revealing the near-complete lack of specific laboratory protocols, whose set-up would undoubtedly improve the quality and reproducibility of scientific research in the field.

Over the years, the study of gene expression has gained importance in many fields of scientific research, providing novel insights about regulatory networks and biological processes. Dye-based Real-time quantitative Polymerase Chain Reaction (RT-qPCR) using dye such as SYBR® Green dye is an economical option for monitoring gene expression which yields reproducible results without the requirement with expensive and labor-intensive fluorescent probe design. Dye-based real time is a sensitive method for the quantification of mRNA in biological samples [5, 6]. Due to its simplicity, rapidity and high specificity, it has become one of the most popular techniques applied for targeted nucleic acid quantification [6]. Despite its wide use and the multiplicity of available protocols, this technique is affected by several problems related to the intrinsic diversity of RNA populations among samples and to the presence of technical errors and experimental biases [6, 7]. To correct these sources of errors, the data collected are usually normalized with one or more internal controls, defined as reference genes whose mRNAs are stably expressed across a broad range of conditions. Although the use of more than one reference gene is usually recommended, their optimal number and combination need to be adequately selected for each experimental condition [6]. Nevertheless, most gene expression studies carried out in molluscs do not use reference genes validated in the species of interest, often relying on genes whose expression is simply assumed to be stable based on data collected from largely divergent vertebrate model species.

The most popular reference genes, used in a wide variety of model organisms, are β-Actin (*Act)*, Glyceraldehyde-3-phosphate dehydrogenase *(Gapdh)* and the 18S ribosomal RNA (*18S*)*Act* can be considered as one of the first internal standards used in gene expression quantification studies, and it is still one of the most widely used internal standards today [8]. Although *Act* is often considered as a single-copy gene, it is part of a multi-gene family that includes several nearly-identical members, which usually allows the design of shared PCR primes [9]. Its mRNA is abundantly expressed in most cell types and encodes a ubiquitous cytoskeleton protein [5]. The *Gapdh* mRNA encodes the glyceraldehyde-3-phosphate dehydrogenase enzyme, which catalyzes an important step in glycolysis. Due to its abundance and ubiquitous expression, it is frequently used as a control in RT-qPCR experiments [5]. Lastly, rRNAs are considered useful internal controls since they are generated by a distinct polymerase (i.e. RNA polymerase 1) [10] and their levels are not expected to vary in a significant manner under conditions affecting the expression of mRNAs [11]. However, a high number of tandemly duplicated identical rRNA genes copies are usually present in eukaryotes, and their abundance depends on genome size [12]. While several studies indicate that *18S rRNA* is a more suitable reference gene than *Gapdh* and *Act* (e.g. [13–15]), others define its use as inappropriate in some cases, such as mammalian cells (e.g. [16, 17]). Since these genes can undergo regulation in response to specific natural or experimental conditions (e.g. *act* mRNA has a cell-cycle dependent expression) a validation process is highly recommended before their inclusion in any panel of reference genes in order to avoid measurement errors [5, 7].

Although a few suitable reference genes for RT-qPCR experiments have been previously identified in mussel [18–20], no specific gene set to be used in different tissues and/or experimental conditions has been described yet. A study carried out on *M. edulis* identified Elongation factor 1 alpha (*Ef1α)* and ribosomal *18S* and *28S* RNAs as the most stable targets across different stages of gametogenesis, while Helicase (Hel) and *Act* were defined as unstable targets [18]. Different combinations of reference genes have been validated for use in *M. galloprovincialis* digestive gland and gills (*Gapdh*, *Ribosomal protein S4* - *Rps4* and *Cytochrome oxidase subunit 1 - Cox1*) and mantle (*Gapdh*, *Rps4* and *Rps27*) following exposure to okadaic acid [19]. A recent study carried out in the golden mussel *Limnoperna fortunei,* highlighted poor stability of gene expression levels in gonads of individuals of different sexes, suggesting the presence of remarkable intra-specific differences [20]. The recently reported widespread hemizygosity of the mussel genome, associated with gene presence-absence variation phenomena, represent another potential issue in the identification of suitable stable reference genes in this species [21].

Some studies in vertebrates (human, mice, zebrafish) and plants (tomato, seaweed, and kiwi) successfully identified housekeeping or reference genes taking advantage of RNA-Seq data [22–26]. To the best of our knowledge, so far this approach has only been used, among molluscs, in scallop due to the unavailability of complete transcriptomic datasets for most species [27].

The aim of the present study was to identify candidate genes suitable as internal references for RT-qPCR experiments in different tissues of the mussel *M. galloprovincialis*. The suitability of *ribosomal protein L14, L32* and *L34* (*rpl14*, *rpl32* and *rpl34*), selected using a transcriptomic approach, was comparatively assessed with seven additional gene targets commonly used in literature and previously used in gene expression studies targeting mussels. We investigated their stability in digestive gland, gills, mantle, gonads and foot in physiological conditions in order to provide a solid basis for future expression studies. We also used the most stable reference genes to assess their reliability in evaluating the expression of four highly expressed tissue-specific genes of interest (GOI) in the mantle and in the digestive gland.

## Results

### Identification of the candidate reference genes from RNA-Seq data

As previously reported by Gerdol et al. [21] *Rpl32*, *Rpl14* and *Rpl34* were identified as the first, third and fourth most stable core genes across 51 RNA-seq datasets from multiple adult tissues and experimental conditions in *M. galloprovincialis*. *Rpl32*, with a stability score equal to 0.24, achieved a mean expression level of 3061.45 TPMs; *Rpl14*, with a stability score equal to 0.25, achieved a mean expression level of 2747.49 TPMs; *Rpl34*, with a stability score equal to 0.27, achieved a mean expression level of 2288.28 TPMs (Figure S1). These three candidate genes were confirmed to lack paralogous gene copies in the *M. galloprovincialis* genome, and in silico primer check ruled out the possibility of non-specific amplification of other genomic regions or transcribed RNAs.

### Expression stability of candidate reference genes

**Fig. 1:**
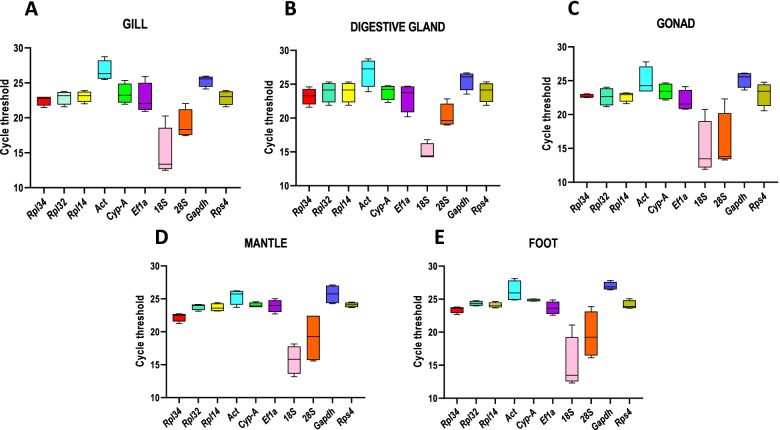
RT-qPCR cycle threshold (Ct) values of candidate reference genes in the different tissues. Ct distribution values of R*pl14, Rpl32, Rpl34, 18S, 28S, Act, Cyp-A, Ef1α, Gapdh* and R*ps4* from gills (**A**), digestive gland (**B**), gonads (**C**), mantle (**D**), foot (**E**) of *M. galloprovincialis.* The distribution is shown by vertical box plot as medians (lines), interquartile range (boxes) and ranges (whiskers)

In this work, we analyzed ten genes, considering three novel candidates selected with a transcriptomic approach (*Rpl14, Rpl32, Rpl34)* and seven genes commonly used as references (*Act, Cyp-A, Ef1α, Gapdh, 18S, 28S, Rps4*), verifying their expression stability in various mussel tissues. The range of cycle threshold (Ct) values was quite similar among samples, with *18S* and *28S* exhibiting the highest range of variation in Ct in all tissues analyzed (Fig. 1, S2).

**Fig. 2:**
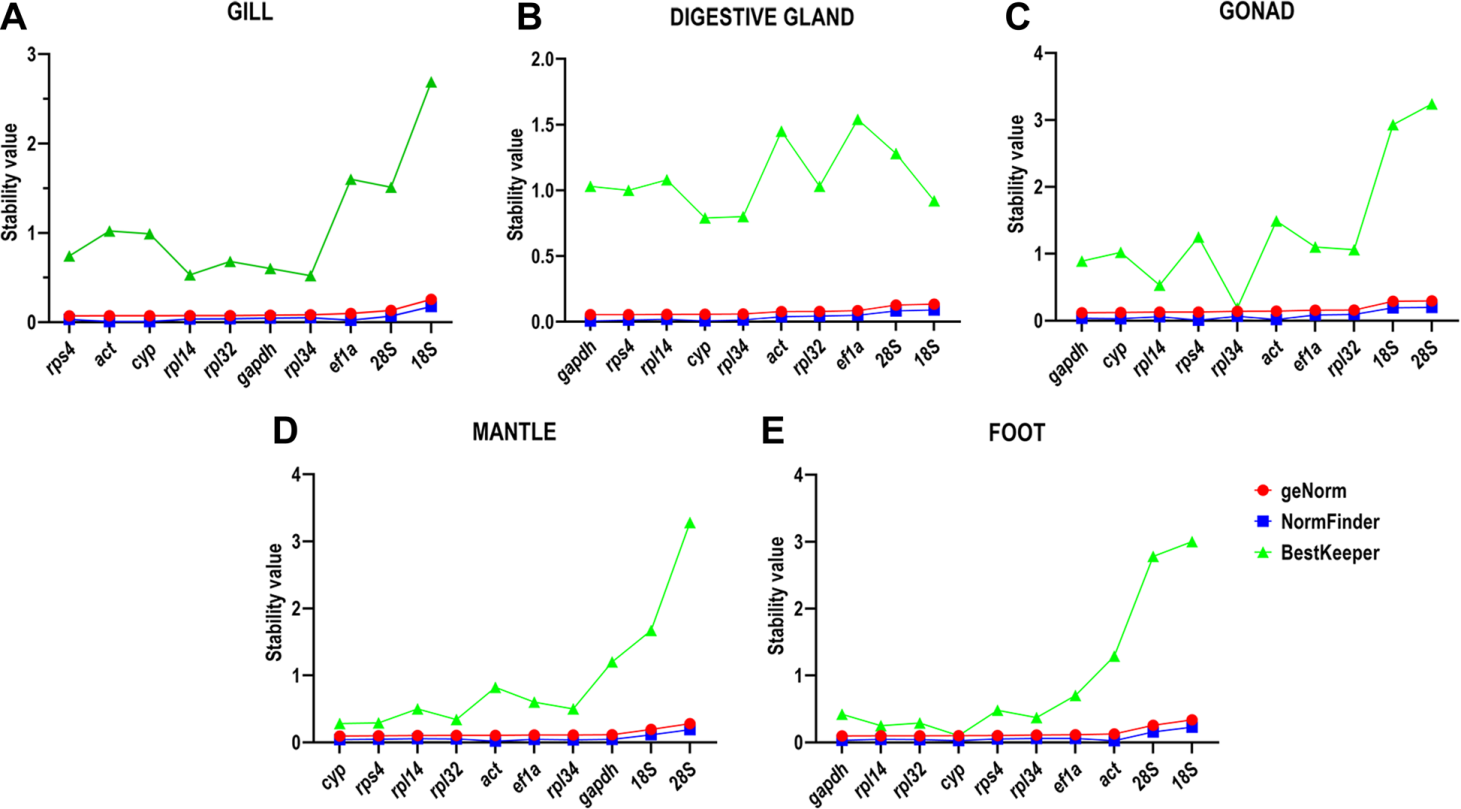
Expression stability of candidate reference genes in the different tissues. Stability of the reference genes was evaluated in Gills (**A**), Digestive Gland (**B**), Gonads (**C**), Mantle (**D**), Foot (**E**) of *M. galloprovincialis* based on geNorm (red circle), NormFinder (blue square) and Bestkeeper (green triangle) analyses of the RT-qPCR experiments. Data is arranged in descending order of comprehensive stability from left to right

An overview of the results obtained from the analysis of the selected reference genes using three different algorithms is presented in Fig. 2. In the gills, *Rps4* was identified as the most stable gene by *geNorm* evaluation, whereas *Act* and *Cyp-A* were selected by the *NormFinder* and *BestKeeper*, respectively. However, all these three genes were generally considered stable by the three algorithms but with a different ranking (Table S1). Interestingly, *geNorm* suggested the use of *Gapdh/Rpl34* as the best combination of reference genes*,* as both showed higher M values than the other analyzed genes (Fig. 2A, Table S1).

In the digestive gland, *Gapdh* and *Rps4* showed the lowest (and identical) M value according to *geNorm;* nevertheless, the algorithm suggested *Rps4/Rpl14* as the best possible combination since the latter gene had an M value slightly higher than *Gapdh* (Table S2). *Gapdh* has ranked first also in the *NormFinder* analysis (Table S2). In addition, both *Rps4* and *Rpl14* displayed good stability values. Conversely, *Gapdh, Rps4* and *Rpl14* were found to be beyond the recommended value (SD < 1) in the BestKeeper analysis, while *Cyp-A* ranked first (Table S2). It is important to note that *Cyp-A* was also identified as a stable gene by the *geNorm* and *NormFinder* analysis (Table S2, Fig. 2B).

The comparison among the different algorithms produced less consistent results in the gonads, with *Gapdh* resulting as the most stable gene by *geNorm*, *Rps4* by *NormFinder* and *Rpl34* by *BestKeeper* (Table S4). In addition, the best combination, according to *geNorm* was *Cyp-A/Gapdh*. All the named genes were generally considered stable by the three algorithms, except for *Rps4* that showed an SD value much higher than recommendations (SD = 1.25) by *BestKeeper* (Table S4, Fig. 2C).

In the mantle, *Cyp-A* showed the highest stability according to both *geNorm* and *BestKeeper*, in contrast with *NormFinder*, which indicated *Act* as the most stable gene (Table S3). Nevertheless, *Cyp-A* and *Act* were considered stable by the three analyses, even though they occupied different positions in the rankings. Furthermore, *geNorm* suggested *Cyp-A/Rpl14* as the best combination of reference genes (Table S3, Fig. 2D).

In the foot, *Gapdh*, *Act* and *Cyp-A* had the best stability values in *geNorm*, *NormFinder* and *BestKeeper* ranking, respectively (Table S5). The best pair of reference genes suggested by *geNorm* was *Rpl14/Rpl32.* As previously discussed for the other tissues, all these genes were considered relatively stable from the different algorithms, with the exception of *act*, which had an SD value higher than 1 (SD = 1.29) (Table S5, Fig. 2E).

**Fig. 3:**
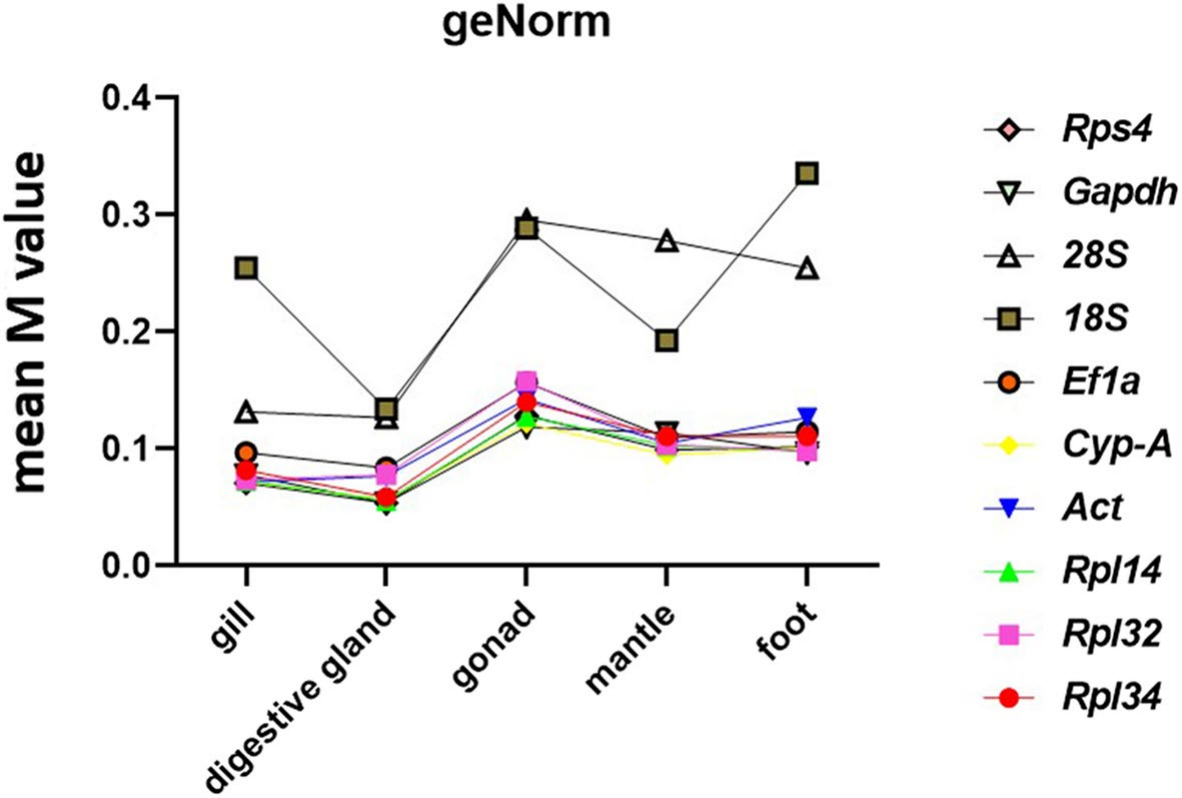
Best candidate reference genes across tissues. Stability of the reference genes was evaluated considering their stability in all the tissues based on geNorm analyses of the RT-qPCR experiments. Data is arranged in descending order of comprehensive stability from left to right

Finally, we reanalyzed the data by considering all the tissues. As previously mentioned for the analysis of single tissues, 18S and 28S were the most variable candidate reference genes across all the tissues (Fig. 3). *NormFinder* defined *Act* and *Cyp-A* as the best gene pair combination.

### Expression level of target genes

We validated the *bonafide* selection of the previously described reference genes for the interpretation expression data of a set of GOI. To this aim, we analyzed the expression of four genes differentially expressed in adult tissues, selected according to their strong specific expression inferred from RNA-seq data in the mantle and digestive gland, respectively. In detail, MGAL10A005692, specifically expressed at high level (averaging ~ 3900 TPM) in the mantle, encodes a low-complexity protein, which bears distant homology with SCO-spondin in the C-terminal region. The second selected mantle-specific GOI, MGAL10A087091, was expressed at similar levels (averaging 3600 TPM) and was orthologous with *M. coruscus* MUSP-3, previously reported to be involved in shell mineralization [28].

On the other hand, MGAL10A040115, a meprin A-like metalloprotease, was one of the most distinctive digestive gland-specific genes in mussel, reaching expression values as high as 100,000 TPM in some samples. The second selected digestive gland GOI, which also displayed very high mean expression values (32,000 TPM) was MGAL10A054097, encoding a protein with multiple ShKT domains.

**Fig. 4:**
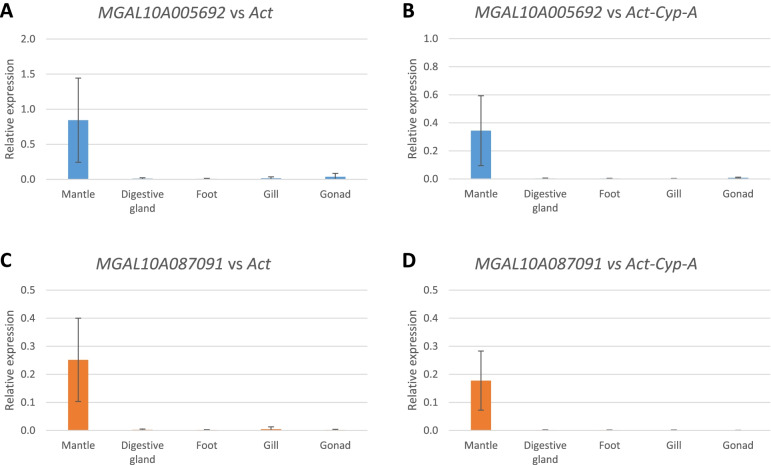
Relative expression of GOI mantle specific.Relative espression was calculate according to the ΔCt methods using *Act* (**A**,**C**) or the Geometric mean of *Act-Cyp-A* (**B**,**D**) as reference. *N* = 3

**Fig. 5:**
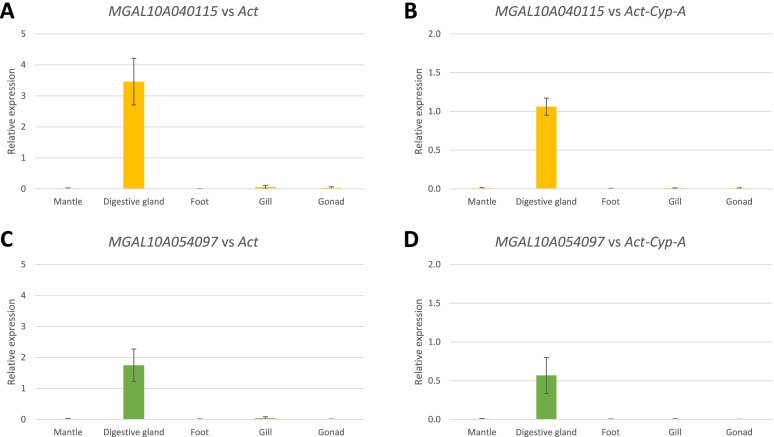
Relative expression of GOI digestive gland specific. Relative expression was calculate according to the ΔCt methods using *Act* (**A**,**C**) or the Geometric mean of *Act-Cyp-A* (**B**,**D**) as reference. *N* = 3

As reported in Fig. 4, the expression profile of MGAL10A005692 and MLGAL10A087091 confirmed the high mantle-specificity indicated by transcriptome data. In particular, the Ct values were higher than 30 in all the tissues other than mantle and the expression of MGAL10A087091 was almost undetectable in gonad and gills (data not shown). The expression profiles of MGAL10A040115 and MGAL10A054097 are reported in Fig. 5. The specificity of these genes for the digestive gland was strongly confirmed, as in all the tissues other than digestive gland the Ct values were higher than 30 (data not shown).

We tested a few other specific GOIs in the digestive gland (MGAL10A065261, MGAL10A066134), in the mantle (MGAL10A006232, MGAL10A059842, MGAL10A079192) and in the foot (MGAL10A033477, MGAL10A064866, MGAL10A070506). However, possibly owing to the low sequence complexity of these gene targets (which often encoded highly repetitive proteins), the efficiency of amplification observed by RT-qPCR data in these cases was very low (data not shown).

For all analyzed GOIs, no significant differences could be evidenced between the use of *Act* as a single reference gene and the use of the *Act-Cyp-A* pair (Figs. 4 and 5). As reported in Figure S3, the use of the last ranking genes, such as 18S and the couple 18S and 28S still allow to detect the tissue specific of the GOIs (i.e MLGAL10A087091 and MGAL10A040115, mantle and digestive gland, respectively) but with an higher standard error.

## Discussion

In this work, we analyzed the stability of ten genes suitable as references, including three novels (*Rpl14, Rpl32, Rpl34)* and several commonly used (*Act, Cyp-A, Ef1α, Gapdh, 18S, 28S, Rps4*) genes within and across different mussel tissues. We first looked at the Ct values, revealing that *Act* was the reference gene showing the highest Ct value among those considered in this study. With this regard, it needs to be specified that primer design targeted one out of the several paralogous actin genes found in the *M. galloprovincialis* genome (sequence ID: AF157491.1), which may not correspond to the most abundant cytoskeletal isoform.

The stability of the suitable reference genes was evaluated using specific algorithms such as *geNorm* [29], *NormFinder* [30] and *BestKeeper* [31]. Despite differences in the ranking of the genes, the results obtained from the three algorithms were quite similar in the different tissues. Most discrepancies were founded in the *BestKeeper* ranking, but all methods were concordant in the identification of unstable genes. Notably, 28S and 18S were the targets with the lowest score in all the tissues according to both *geNorm* and *NormFinder*, and 18S was one the most stable reference only in the DG according to *BestKeeper* (Fig. 2). The poor stability of rRNA expression may have multiple explanations, which include the presence of inter-individual variability in the number of rRNA gene copies present in individual genomes and a different level of DNA methylation of rRNA gene clusters. Although this has been previously demonstrated in human [32, 33], to the best of our knowledge no study has ever been carried in bivalves to clarify this aspect. Nevertheless, the widespread hemizygosity and gene presence/absence variation phenomena found in mussel and other bivalves [34] may provide an explanation for the poor stability of expression of 18S and 28S rRNA genes in *M. galloprovincialis*.

As shown in Tables S1, S2, S3, S4 and S5, all the other genes were considered relatively stable with *geNorm* and *NormFinder*, even with differences in the specific ranking. On the other hand, the *BestKeeper* analysis calculated a SD value below the desired threshold for some suitable references. These include *Act* (in gills, digestive gland, gonads and foot), *Ef1α* (in gills, digestive gland and gonad), *18S* (in gills, digestive glands, gonads, foot), *Rsp4* (in digestive gland and gonads), *Gapdh* (in digestive gland and mantle), *Rpl32* (in digestive gland and gonad), *Rpl14* (in digestive gland) and *Cyp-A* (in gonads).

Finally, the analyses carried out revealed some discrepancies between RNA-seq and RT-qPCR expression data, such as the low performance of the three newly selected reference genes compared to those traditionally used in literature. This could definitely become a point of interest, not only for mussels studies but possibly for many other non-model marine organisms for which no specific reference genes have been identified and validated yet. Although RT-qPCR approaches are commonly used to confirm the results of differential gene expression analyses carried out on an “omic” scale, the data obtained with the two methodologies are not always concordant. Such differences can be explained by multiple technical and biological factors, which include, among the others, the different dynamic range of the two methods and the dependence of RT-qPCR on appropriate primer design [35]. One crucial point that could mitigate these potential issues, in particular in non-model organisms with a complex genomic background such as *M. galloprovincialis*, could be the choice of reference genes showing high stability in the biological context of interest. Other “bench tips”, such as those part of the MIQE guidelines [6], may further improve the application of RT-qPCR analyses to non-model marine species and allow their comparison with the results obtained with “omic” approaches.

## Conclusions

In the present study, we analyzed the expression profiles of ten candidate genes from *M. galloprovincialis* in physiological state to assess their potential use as references in RT-qPCR experiments. Normalization strategies are required to correct experimental errors such as variations in extraction yield, reverse-transcription yield and efficiency of amplification [6]. Although the calculation of the expression levels of genes of interest commonly relies on the use of reference genes as internal controls for normalization, their reliability must be experimentally validated for each experimental design [6]. The analyses conducted with *geNorm* [29], *NormFinder* [30] and *BestKeeper* [31] allowed us to identify the best candidate genes to be used in different adult mussel tissues, as well as the most suitable pair of references to be used for comparisons among tissues (*Act/Cyp-A*). Remarkably, the *18S* and *28S* rRNA genes, which use as references is widespread in literature, were in all cases the more unstable among the ten candidate reference genes tested. We therefore suggest that RT-qPCR analyses in *M. galloprovincialis* should rely on the use of different nuclear reference genes, to be carefully selected based on the tissue of interest and the experimental conditions.

## Methods

### Sample collection

For the validation of reference genes, adult *M. galloprovincialis* mussels (5.0 ± 1.0 cm shell length) were sampled from the Bay of Naples (Italy) in autumn 2019. In the laboratory, specimens were dissected to separate digestive gland, gills, mantle, gonad and foot. Tissue samples were immediately weighed and stored at − 80 °C until further analysis.

### In silico identification of candidate stable reference genes and GOI

“A total of 51 different RNA-seq dataset were mapped to the *M. galloprovincialis* reference assembly. These datasets derive from multiple previously published studies, carried out by different research teams and taking into account mussels sampled in different geographical locations [36–40]. These transcriptomic data refers to multiple tissues, subject to different experimental conditions (see Table S6)”.. Only *core* genes (i.e. those invariably present in all individuals) were considered, whereas *dispensable* genes (i.e. those subject to presence-absence variation) were discarded. Mapping was carried out with the CLC Genomics Workbench v. 20 (Qiagen, Hilden, Germany), setting the *length fraction* and *similarity fraction* parameters to 0.75 and 0.98, respectively. Digital read counts were used to computing gene expression levels as Transcript Per Million (TPM) [41]. The selection of candidate stable reference gene followed an approach similar to the one described below for BestKeeper (see Results paragraph). The mean expression value was calculated for each *core* gene across all 51 samples. All TPM expression values, for each gene and dataset, were subsequently transformed by dividing them by the mean value, obtaining a distribution of all records centred on 1. The calculation of standard deviation of the standardized gene expression values allowed to evaluate gene expression stability, with the most and least stable genes being identified by low and high SD values, respectively. The four GOIs (two for the digestive gland and two for the mantle tissue) were selected based on the following criteria: *(i)* inclusion in the *M. galloprovincialis* core gene set, indicating presence in all individuals; *(ii)* absence of paralogous genes, to avoid any chance of non-specific cross-amplification; (iii) high expression in the tissue of interest (i.e. > = 500 TPM), as evaluated by the analysis of the available RNA-seq datasets (Table S6); *(iii)* highly specific tissue expression, defined as a fold change > = 10 in the pairwise comparisons between the expression levels observed in digestive gland/mantle and all the other tissues; *(iv)* a gene architecture compatible with the design of primer pairs able to discriminate between specific cDNA amplification signals and genomic DNA contamination.

### Total RNA extraction and cDNA synthesis

Up to 100 mg of each tissue were lysed and total RNA was processed with *SV Total RNA Isolation System* kit (Promega) according to the manufacturer’s instructions. For all RNA samples, the absence of DNA contamination was tested by performing PCR with *ef1α* primers designed within an exon (for sequence see Table 1 for *ef1α-*g). Concentration and purity of all RNA samples were measured using a Nanodrop ND1000 spectrophotometer. RNA integrity was evaluated through 1.5% agarose gel electrophoresis. Total RNA (0.3 μg) from each tissue was reverse transcribed using the *iScript RT Supermix* (Bio-rad) following the manufacturer’s protocol and stored at − 20 °C. Subsequently cDNA was diluted with H_2_O prior to use in RT-qPCR experiments.

**Table 1:** Primer sequences

Gene		Primer sequence 5′-3′	Amplicon	Intron	Refs	E
***Ef1α-g***	F	TTTCTGGATGGCACGGAGAC	150 bp	No		
	R	CTGTGGGTCTTGATGGTGGG				
***Rpl34***	F	ACACAAAGAAGGCAGGTGCAG	136 bp	Yes		2.0
	R	GACCCTCCATAAGCTCTGGTTAC				
***Rpl32***	F	CACTTGATGCCTGATGGTTTCC	143 bp	Yes		2.0
	R	GCACGCTCAACGATTTCTTTCC				
***Rpl14***	F	CAATGCCAAGATCCCTCCGTAC	167 bp	Yes		2.0
	R	AGCTTGCTTGGCCTTCATCAG				
***Act***	F	ATGCTCCAAGAGCCGTGTTTC	114 bp	Yes		1.9
	R	CTCTCTTGCTCTGGGCTTCATC				
***Cyp-A***	F	GGAGTTGAGAGCTGATGTTGTACC	100 bp	Yes		1.9
	R	TCGGTGGAATTTGCTGTCTTGG				
***Ef1α***	F	CCAGTGGCAAGACCTTATTCGAG	183 bp	Yes		2.0
	R	TGGCTGGAGCAAAGGTAACAAC				
***18S***	F	AGAAACGGCTACCACATCCA	160 bp	Yes		2.0
	R	TGCCCTCCAATAGATCCTCG				
***28S***	F	AGTGCACTTTCCTCGGGTAG	123 bp	Yes		2.1
	R	GACGAGTCGACACTAGACGG				
***Gapdh***	F	AGGAATGGCCTTCAGGGTAC	114 bp	Yes	[19]	2.1
	R	TCAGATGCTGCTTTAATGGCTG				
***Rps4***	F	TGGGTTATCGAGGGCGTAG	121 bp	Yes	[19]	1.9
	R	TCCCTTAGTTTGTGAGGACCTG				
***MGAL10A040115***	F	TGCCAACCACAGACTTCTCT	167 bp	Yes		1.9
	R	CTTTGCGTGGTTGGAAGTCA				
***MGAL10A054097***	F	AACAACCCCAGTACCACCTT	212 bp	Yes		1.9
	R	TCTTTAATGGCAAGCTGGGC				
***MGAL10A087091***	F	GACAACGGCCAAGGAATAGC	141 bp	Yes		2.0
	R	TTCGTTCCTGGACCACTCTG				
***MGAL10A005692***	F	GCAGGTGTTGTTGTCATGGT	156 bp	Yes		2.1
	R	AGAGAGAGCTTGGTTCGTGT				

*F* Forward, *R* Reverse, amplicon size in bp, presence of an intron within the amplified region and calculated reaction efficiencies (E) for RT-qPCR experiments

### Primer design

Ten putative reference genes: *Act*, *Cyp-A*, *Ef1α*, *18S*, *28S*, *Rpl34*, *Rpl32*, *Rpl14*, *Gapdh* and *Rps4* and four GOI (MGAL10A005692, MGAL10A087091, MGAL10A040115 and MGAL10A054097) were selected. Primers are listed in Table 1.

Genomic sequences were retrieved from NCBI or from the reference genome assembly [21] and gene structure was predicted using Splilign (https://www.ncbi.nlm.nih.gov/sutils/splign/splign.cgi). RT-qPCR primers were manually designed in adjacent exons and verified using Primer blast (https://www.ncbi.nlm.nih.gov/tools/primer-blast/index.cgi?LINK_LOC=BlastHome).

The sequences of *Gapdh* and *Rsp4* primers were retrieved from the work of Martinez-Escauriaza et al. (2018) and the presence of a spanning intron was verified as described above.

Previously generated *M. galloprovincialis* whole genome sequencing data [21] were used to confirm that all selected target genes were not subject to presence-absence variation. At the same time, primer specificity was assessed in silico by BLASTn against the reference genome assembly, verifying the absence of paralogous genes and significant non-specific matches between the forward and reverse primers and non-target genomic regions.

The specificity of amplicons was confirmed by the presence of a single band of the expected size on 2% agarose gel. Finally, the PCR products were purified using the *illustra GFX PCR DNA and Gel Band Purification* kit (GE Healthcare) following the manufacturer’s instructions and their identity was confirmed by sequencing.

### Real time qPCR

Diluted cDNA from the different tissues was used as template in a reaction containing a final concentration of 0.5 μM for each primer and 1 × FastStart SYBR Green master mix (total volume of 10 μl). PCR amplifications were performed in triplicate using the ViiA™ 7 Real-Time PCR System (Applied Biosystems) applying the following thermal profile: 95 °C for 10 min, one cycle for cDNA denaturation; 95 °C for 35 s, 58 °C for 30s and 72 °C for 40s, 40 cycles for amplification; one cycle for melting curve analysis, to verify the presence of a single product. Each assay included a no-template control for each primer pair. We used tissues from four and three different animals to test reference genes stability and GOI expression level, respectively.

The corresponding real-time PCR efficiency (E)- reported in Table 1 can be obtained in two ways. It can be computed either as sample-specific [42], or as factor specific [43] metric according to the eq. E = 10^–1/slope^. The slope of linear regression model fitted over log-transformed data of five serially diluted input cDNA concentrations plotted against the corresponding CT values [43, 44]. The maximal efficiency of PCR is E = 2, in the case where every single template is replicated in each cycle; the minimal value is E = 1, corresponding to no replication.

### Analysis of gene expression stability

The gene expression stability of the candidate genes was evaluated using *geNorm* [29], *NormFinder* [30] and *BestKeeper* [31]. For each algorithm, a stepwise exclusion method was applied in order to rank the selected genes according to their expression stability.

The *geNorm*-Based Analysis of candidate reference genes aims to select suitable references considering those displaying minimal variation across different biological conditions. Ideally, the normalized expression of GOI is obtained using the geometric mean of the optimal number of references selected in the analysis. This analysis renders a ranking of the tested genes based on their stability measure (M), determining the two most stable references or even a combination of multiple stable genes to be used for normalization. The M value results from the mean pairwise variation between every single gene compared to all other tested candidates. The algorithm discards the gene with the highest M and recalculates a new score for the remaining genes in a stepwise manner. Thus, references rank according to their M, from the most (lowest M values) to the least stable (highest M values) [29].

The *NormFinder* approach identifies the optimal reference among a set of candidates according to their expression stability in a given sample set and a given experimental design. This approach is based on a two-way ANOVA and provides a stability value for each reference analyzed, which is a direct measure of the estimated expression variation, which allows the user to evaluate the systematic error introduced when using the gene for normalization [30].

*BestKeeper* performs the descriptive statistic for each candidate, calculates the expression levels of the candidate references and estimates their expression stability based on the inspection of calculated variations (SD values). The candidates are then ordered from the most stably expressed, with the lowest variation, to the least stable one, with the highest mobility, according to the variability observed. Any evaluated gene with an SD higher than 1 (= starting template variation by the factor 2) is considered inconsistent [31].

### Expression analysis of target genes

The relative expression level of each GOI was calculated using the most stable reference gene/genes identified across all tissues by *NormFinder*. In detail, relative expression levels were calculated as previously described [45], applying the ΔCt method, considering the E = 2 and using either a single reference or the geometric mean of the best pair of references for normalization. Whenever no amplification signal could be obtained for a GOI in a given tissue to allow calculation, we arbitrarily assigned to these samples a Ct value = 40.

## Supplementary Information

**Additional file 1.**

## Data Availability

All data analysed during this study are included in the published article: Gerdol M, Moreira R, Cruz F, Gómez-Garrido J, Vlasova A, Rosani U, Venier P, Naranjo-Ortiz MA, Murgarella M, Greco S, Balseiro P, Corvelo A, Frias L, Gut M, Gabaldón T, Pallavicini A, Canchaya C, Novoa B, Alioto TS, Posada D, Figueras A. Massive gene presence-absence variation shapes an open pan-genome in the Mediterranean mussel. Genome Biol. 2020 Nov 10;21(1):275. doi: 10.1186/s13059-020-02180-3

## Abbreviations

Ct: Cycle threshold
GOI: Gene Of Interest
M: Stability Measure
TPM: Transcript Per Million
