# Supplementary material for: Comparative analysis of novel and common reference genes in adult tissues of the mussel Mytilus galloprovincialis
Source: BMC Genomics. 2022 May 6;23:349. doi: 10.1186/s12864-022-08553-1 (PMC9077915; doi:10.1186/s12864-022-08553-1)
Supplement: Supplementary file 1 — Additional file 1. [file 12864_2022_8553_MOESM1_ESM.docx]

**Supplementary figures and tables**


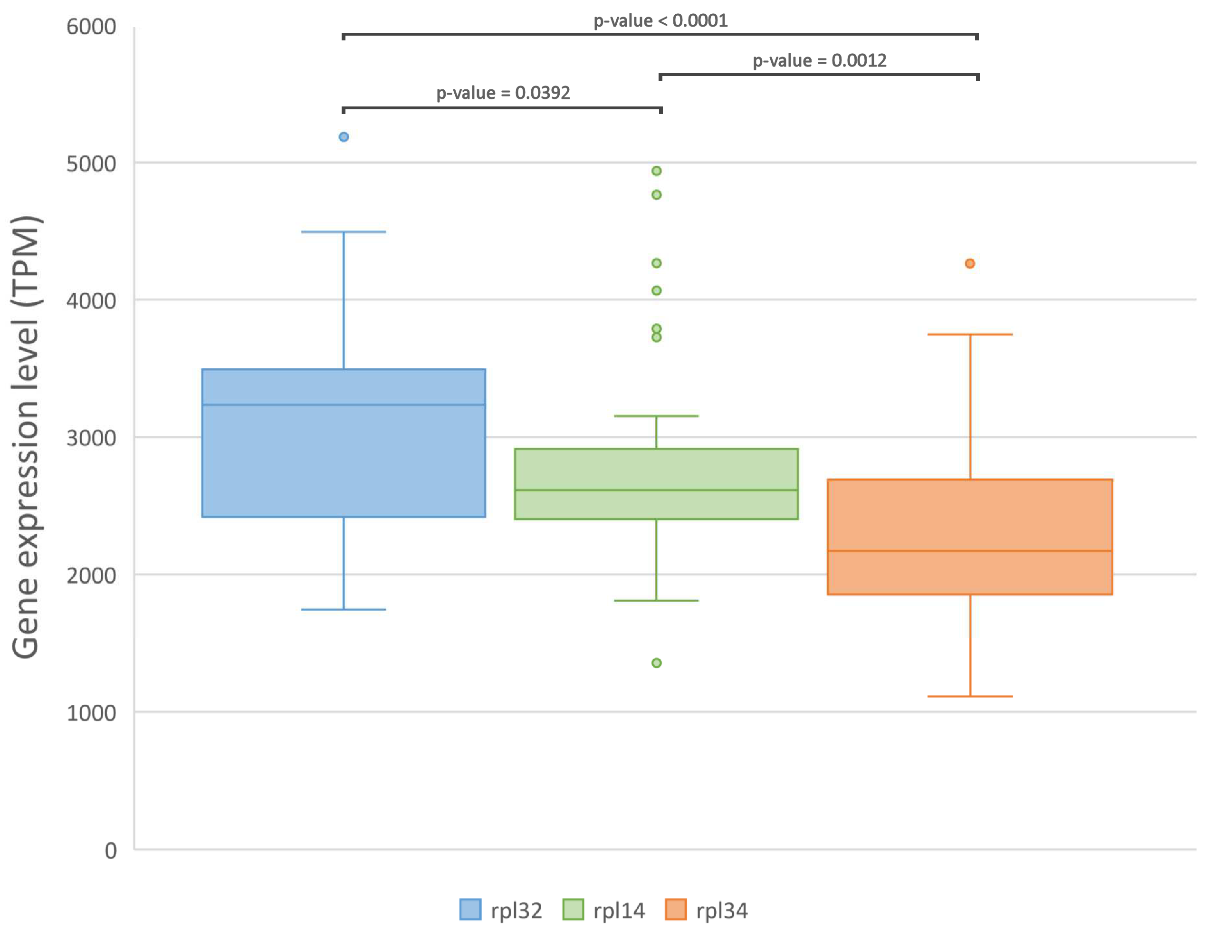


**Figure S1** *Expression levels of rpl32, rpl14 and rpl34 genes across 51 RNA-seq datasets*. Gene expression levels are shown as Transcripts Per Million (TPM). The graphs indicate the median and interquartile ranges. The p-values resulting from pairwise comparisons of gene expression levels among the three genes (based on an unpaired T-test) are indicated.

**Figure S2** *RT-qPCR cycle threshold (Ct) values of candidate reference genes among different tissues.* Ct distribution values of *rpl14, rpl32, rpl34, 18S, 28S, act, cyp, ef1α, gapdh* and *rps4* from Gilles, Digestive Gland, Gonads, Mantle, Foot of *M. galloprovincialis.* The distribution is shown by vertical box plot as medians (lines), interquartile range (boxes) and ranges (whiskers).


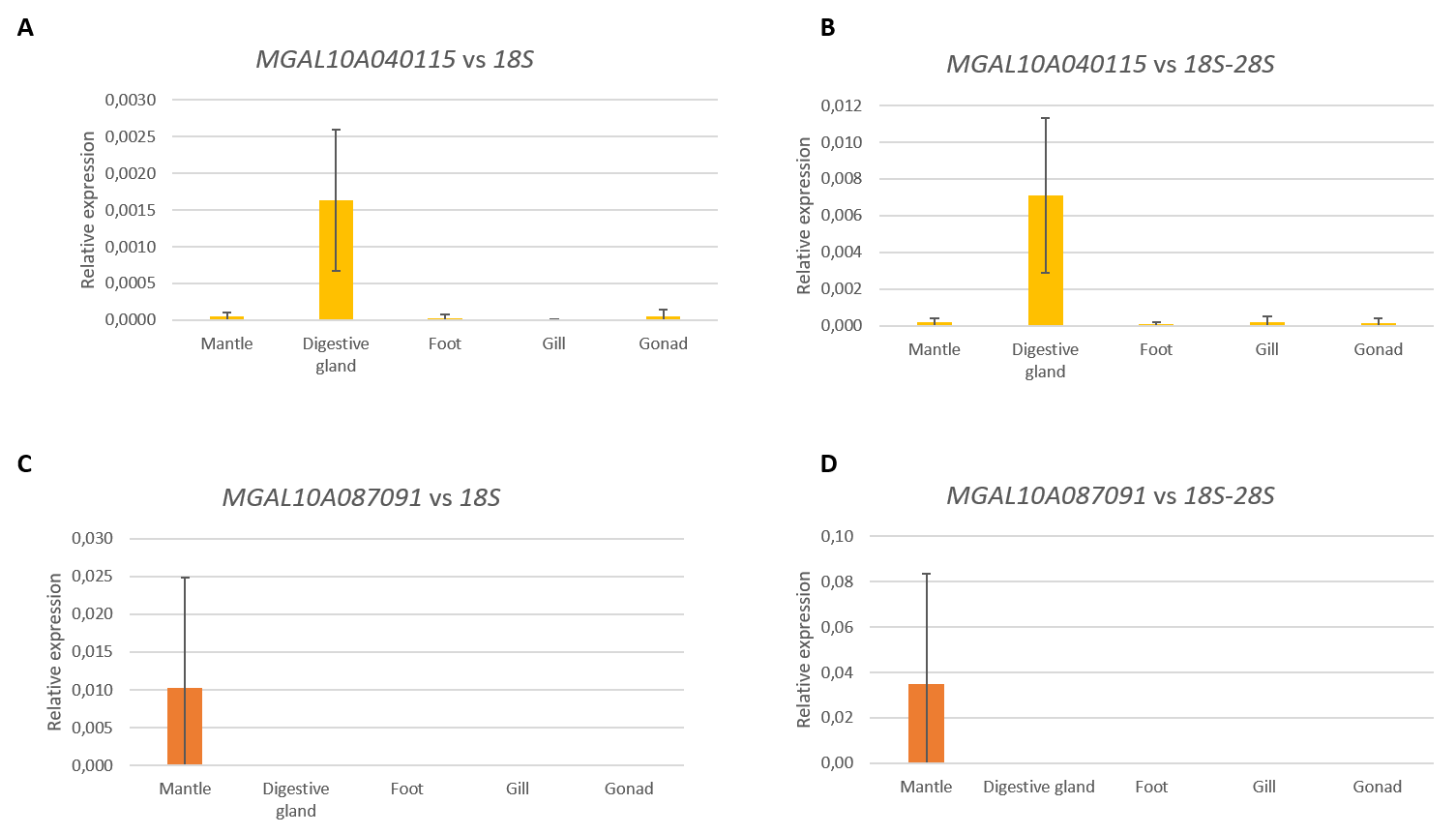


**Figure S3** *Relative expression of GOI using not the worst ranked candidate reference genes.* Relative expression was calculate according to the ΔCt methods using *18S* (A,C) or the Geometric mean of *18S-28S* (B,D) as reference. N=3.

**Table S1** *Summary of results for the most stable candidate reference genes in the gill.*

| **Rank** | ***geNorm: mean M value*** | | ***NormFinder: stability value*** | | ***BestKeeper: SD*** | |
| --- | --- | --- | --- | --- | --- | --- |
| GL |  |  |  |  |  |  |
| 1 | ***Rps4*** | **0.071** | ***Act*** | **0.006** | ***Rpl34*** | **0.52** |
| 2 | ***Act*** | **0.072** | ***Cyp-A*** | **0.006** | ***Rpl14*** | **0.53** |
| 3 | ***Cyp-A*** | **0.072** | ***Ef1a*** | **0.021** | ***Gapdh*** | **0.60** |
| 4 | ***Rpl14*** | **0.073** | ***Rps4*** | **0.031** | ***Rpl32*** | **0.68** |
| 5 | ***Rpl32*** | **0.074** | ***Rpl14*** | **0.035** | ***Rps4*** | **0.74** |
| 6 | ***Gapdh*** | **0.078** | ***Rpl32*** | **0.038** | ***Cyp-A*** | **0.99** |
| 7 | ***Rpl34*** | **0.082** | ***Gapdh*** | **0.045** | *Act* | 1.02 |
| 8 | ***Ef1a*** | **0.097** | ***Rpl34*** | **0.050** | *28S* | 1.51 |
| 9 | ***28S*** | **0.132** | ***28S*** | **0.067** | *Ef1a* | 1.60 |
| 10 | ***18S*** | **0.255** | ***18S*** | **0.175** | *18S* | 2.69 |

Candidate reference genes for normalization were identified according to *BestKeeper* (Pfaffl *et al.*, 2004), *geNorm* (Vandesompele *et al.*, 2002) and *NormFinder* (Andersen *et al.*, 2004). Values within the recommended thresholds are in bold. Black rectangles highlight the best combination of genes for each tissue according to *geNorm* (Vandesompele *et al.*, 2002).

**Table S2** *Summary of results for the most stable candidate reference genes in the digestive gland.*

| **Rank** | ***geNorm: mean M value*** | | ***NormFinder: stability value*** | | ***BestKeeper: SD*** | |
| --- | --- | --- | --- | --- | --- | --- |
| DG |  |  |  |  |  |  |
| 1 | ***Gapdh*** | **0.054** | ***Gapdh*** | **0.006** | ***Cyp-A*** | **0.79** |
| 2 | ***Rps4*** | **0.054** | ***Cyp-A*** | **0.007** | ***Rpl34*** | **0.80** |
| 3 | ***Rpl14*** | **0.056** | ***Rps4*** | **0.013** | ***18S*** | **0.92** |
| 4 | ***Cyp-A*** | **0.057** | ***Rpl34*** | **0.014** | ***Rps4*** | **1.00** |
| 5 | ***Rpl34*** | **0.059** | ***Rpl14*** | **0.019** | *Gapdh* | 1.03 |
| 6 | ***Act*** | **0.077** | ***Act*** | **0.038** | *Rpl32* | 1.03 |
| 7 | ***Rpl32*** | **0.078** | ***Rpl32*** | **0.043** | *Rpl14* | 1.08 |
| 8 | ***Ef1a*** | **0.084** | ***Ef1a*** | **0.049** | *28S* | 1.28 |
| 9 | ***28S*** | **0.127** | ***28S*** | **0.082** | *Act* | 1.45 |
| 10 | ***18S*** | **0.134** | ***18S*** | **0.089** | *Ef1a* | 1.54 |

Candidate reference genes for normalization were identified according to *BestKeeper* (Pfaffl *et al.*, 2004), *geNorm* (Vandesompele *et al.*, 2002) and *NormFinder* (Andersen *et al.*, 2004). Values within the recommended thresholds are in bold. Black rectangles highlight the best combination of genes for each tissue according to *geNorm* (Vandesompele *et al.*, 2002).

**Table S3** *Summary of results for the most stable candidate reference genes in the mantle.*

| **Rank** | ***geNorm: mean M value*** | | ***NormFinder: stability value*** | | ***BestKeeper: SD*** | |
| --- | --- | --- | --- | --- | --- | --- |
| M |  |  |  |  |  |  |
| 1 | ***Cyp-A*** | **0.095** | ***Act*** | **0.018** | ***Cyp-A*** | **0.28** |
| 2 | ***Rps4*** | **0.099** | ***Rpl34*** | **0.036** | ***Rps4*** | **0.29** |
| 3 | ***Rpl14*** | **0.103** | ***Cyp-A*** | **0.041** | ***Rpl32*** | **0.34** |
| 4 | ***Rpl32*** | **0.104** | ***Ef1a*** | **0.044** | ***Rpl14*** | **0.50** |
| 5 | ***Act*** | **0.105** | ***Gapdh*** | **0.045** | ***Rpl34*** | **0.50** |
| 6 | ***Ef1a*** | **0.11** | ***Rps4*** | **0.047** | ***Ef1a*** | **0.60** |
| 7 | ***Rpl34*** | **0.111** | ***Rpl32*** | **0.050** | ***Act*** | **0.82** |
| 8 | ***Gapdh*** | **0.114** | ***Rpl14*** | **0.055** | *Gapdh* | 1.20 |
| 9 | ***18S*** | **0.193** | ***18S*** | **0.113** | *18S* | 1.67 |
| 10 | ***28S*** | **0.278** | ***28S*** | **0.189** | *28S* | 3.28 |

Candidate reference genes for normalization were identified according to *BestKeeper* (Pfaffl *et al.*, 2004), *geNorm* (Vandesompele *et al.*, 2002) and *NormFinder* (Andersen *et al.*, 2004). Values within the recommended thresholds are in bold. Black rectangles highlight the best combination of genes for each tissue according to *geNorm* (Vandesompele *et al.*, 2002).

**Table S4** *Summary of results for the most stable candidate reference genes in the gonad.*

| **Rank** | ***geNorm: mean M value*** | | ***NormFinder: stability value*** | | ***BestKeeper: SD*** | |
| --- | --- | --- | --- | --- | --- | --- |
| GO |  |  |  |  |  |  |
| 1 | ***Gapdh*** | **0.119** | ***Rps4*** | **0.007** | ***Rpl34*** | **0.19** |
| 2 | ***Cyp-A*** | **0.122** | ***Act*** | **0.017** | ***Rpl14*** | **0.53** |
| 3 | ***Rpl14*** | **0.128** | ***Cyp-A*** | **0.028** | ***Gapdh*** | **0.89** |
| 4 | ***Rps4*** | **0.129** | ***Gapdh*** | **0.035** | *Cyp-A* | 1.02 |
| 5 | ***Rpl34*** | **0.140** | ***Rpl14*** | **0.058** | *Rpl32* | 1.06 |
| 6 | ***Act*** | **0.143** | ***Rpl34*** | **0.065** | *Ef1a* | 1.10 |
| 7 | ***Ef1a*** | **0.157** | ***Ef1a*** | **0.085** | *Rps4* | 1.25 |
| 8 | ***Rpl32*** | **0.158** | ***Rpl32*** | **0.093** | *Act* | 1.49 |
| 9 | ***18S*** | **0.289** | ***18S*** | **0.191** | *18S* | 2.93 |
| 10 | ***28S*** | **0.296** | ***28S*** | **0.198** | *28S* | 3.24 |

Candidate reference genes for normalization were identified according to *BestKeeper* (Pfaffl *et al.*, 2004), *geNorm* (Vandesompele *et al.*, 2002) and *NormFinder* (Andersen *et al.*, 2004). Values within the recommended thresholds are in bold. Black rectangles highlight the best combination of genes for each tissue according to *geNorm* (Vandesompele *et al.*, 2002).

**Table S5** *Summary of results for the most stable candidate reference genes in the foot.*

| **Rank** | ***geNorm: mean M value*** | | ***NormFinder: stability value*** | | ***BestKeeper: SD*** | |
| --- | --- | --- | --- | --- | --- | --- |
| F |  |  |  |  |  |  |
| 1 | ***Gapdh*** | **0.097** | ***Act*** | **0.025** | ***Cyp-A*** | **0.10** |
| 2 | ***Rpl14*** | **0.098** | ***Cyp-A*** | **0.026** | ***Rpl14*** | **0.25** |
| 3 | ***Rpl32*** | **0.098** | ***Gapdh*** | **0.031** | ***Rpl32*** | **0.29** |
| 4 | ***Cyp-A*** | **0.101** | ***Rpl32*** | **0.040** | ***Rpl34*** | **0.37** |
| 5 | ***Rps4*** | **0.102** | ***Rpl14*** | **0.045** | ***Gapdh*** | **0.42** |
| 6 | ***Rpl34*** | **0.111** | ***Rps4*** | **0.050** | ***Rps4*** | **0.48** |
| 7 | ***Ef1a*** | **0.115** | ***Ef1a*** | **0.058** | ***Ef1a*** | **0.70** |
| 8 | ***Act*** | **0.127** | ***Rpl34*** | **0.059** | *Act* | 1.29 |
| 9 | ***28S*** | **0.255** | ***28S*** | **0.157** | *28S* | 2.78 |
| 10 | ***18S*** | **0.336** | ***18S*** | **0.227** | *18S* | 3.00 |

Candidate reference genes for normalization were identified according to *BestKeeper* (Pfaffl *et al.*, 2004), *geNorm* (Vandesompele *et al.*, 2002) and *NormFinder* (Andersen *et al.*, 2004). Values within the recommended thresholds are in bold. Black rectangles highlight the best combination of genes for each tissue according to *geNorm* (Vandesompele *et al.*, 2002).

**Table S6** *List of SRA accession IDs of the RNA-seq experiments analyzed in this study.*

| **SRA Accession** | **Tissue** |
| --- | --- |
| SRX126945 | digestive gland |
| SRX126946 | digestive gland |
| SRX126947 | digestive gland |
| SRX126948 | digestive gland |
| SRX126949 | digestive gland |
| SRX126950 | digestive gland |
| SRX3198556 | whole body |
| SRX565225 | whole body |
| SRX565226 | whole body |
| SRX565227 | whole body |
| SRX565228 | whole body |
| SRX565229 | whole body |
| SRX565230 | whole body |
| SRX386628 | hemocytes |
| SRX389338 | hemocytes |
| SRX389462 | mantle |
| SRX389463 | mantle |
| SRX389464 | posterior adductor muscle |
| SRX389465 | posterior adductor muscle |
| SRX389466 | gills |
| SRR2392495 | anterior mantle |
| SRR2392762 | mid mantle |
| SRR2409049 | posterior mantle |
| ERR4296980 | digestive gland |
| ERR4296979 | gills |
| SRX4059480 | hemocytes |
| SRX4059481 | hemocytes |
| SRX4059482 | hemocytes |
| SRX4059483 | hemocytes |
| SRX4059484 | hemocytes |
| SRX4059485 | hemocytes |
| SRX4059486 | hemocytes |
| SRX4059487 | hemocytes |
| SRX4059488 | hemocytes |
| SRX4059489 | hemocytes |
| SRX4059490 | hemocytes |
| SRX4059491 | hemocytes |
| SRX4059492 | hemocytes |
| SRX4059493 | hemocytes |
| SRX4059494 | hemocytes |
| SRX4059495 | hemocytes |
| SRX4059496 | hemocytes |
| SRX4059497 | hemocytes |
| SRX4059498 | hemocytes |
| SRX4059499 | hemocytes |
| SRX4059500 | hemocytes |
| SRX4059501 | hemocytes |
| SRX4059502 | hemocytes |
| SRX4059503 | hemocytes |
